# A Muscle-Centric Perspective on Intermittent Fasting: A Suboptimal Dietary Strategy for Supporting Muscle Protein Remodeling and Muscle Mass?

**DOI:** 10.3389/fnut.2021.640621

**Published:** 2021-06-09

**Authors:** Eric Williamson, Daniel R. Moore

**Affiliations:** Faculty of Kinesiology and Physical Education, University of Toronto, Toronto, ON, Canada

**Keywords:** intermittent fasting, muscle protein metabolism, dietary protein, muscle mass, weight loss, muscle protein synthesis/breakdown, lean body mass, time-restricted eating

## Abstract

Muscle protein is constantly “turning over” through the breakdown of old/damaged proteins and the resynthesis of new functional proteins, the algebraic difference determining net muscle gain, maintenance, or loss. This turnover, which is sensitive to the nutritional environment, ultimately determines the mass, quality, and health of skeletal muscle over time. Intermittent fasting has become a topic of interest in the health community as an avenue to improve health and body composition primarily via caloric deficiency as well as enhanced lipolysis and fat oxidation secondary to attenuated daily insulin response. However, this approach belies the established anti-catabolic effect of insulin on skeletal muscle. More importantly, muscle protein synthesis, which is the primary regulated turnover variable in healthy humans, is stimulated by the consumption of dietary amino acids, a process that is saturated at a moderate protein intake. While limited research has explored the effect of intermittent fasting on muscle-related outcomes, we propose that infrequent meal feeding and periods of prolonged fasting characteristic of models of intermittent fasting may be counter-productive to optimizing muscle protein turnover and net muscle protein balance. The present commentary will discuss the regulation of muscle protein turnover across fasted and fed cycles and contrast it with studies exploring how dietary manipulation alters the partitioning of fat and lean body mass. It is our position that intermittent fasting likely represents a suboptimal dietary approach to remodel skeletal muscle, which could impact the ability to maintain or enhance muscle mass and quality, especially during periods of reduced energy availability.

## Introduction

Skeletal muscle's central role is the production of contractile force. However, this tissue also serves as the primary site of postprandial glucose disposal (1) and is the largest contributor to resting energy expenditure (2), which collectively positions it as a vital tissue for the maintenance of health and function. Muscle is a dynamic tissue in a constant state of turnover as characterized by rates of muscle protein synthesis (MPS) and muscle protein breakdown (MPB). These processes are responsive to nutrients and contractile activity with changes in MPS and MPB ultimately influencing muscle tissue mass, quality, and health, all of which can influence physical performance (3), injury prevalence (4), and disease risk and/or progression in clinical populations (5).

MPB, which is primarily influenced by the suppressive effect of insulin (6), serves to eliminate old, damaged, mutated and/or redundant proteins through breakdown into their constituent amino acids (AA) (7). These liberated AA enter the muscle's free intracellular pool whereby they may serve as a fuel source (e.g., oxidative phosphorylation) or precursors to be recycled back into protein synthesis. Intramuscular free AA can also be released into circulation to be used by other tissues for synthesis, oxidation or as substrates for gluconeogenesis or ketogenesis (e.g., in the liver), the latter of which is irreversible and contributes to net AA loss. The prevailing view is that MPB plays a relatively minor role in the regulation of muscle mass in healthy humans (7), although whole body protein breakdown that is influenced by higher turning over non-muscle protein pools may play a greater role in whole body net protein balance (8).

MPS is the sequencing of individual AA, made available through protein breakdown or exogenous sources (e.g., digestion and absorption of dietary protein/AA), into polypeptide chains that form the functional protein of muscle tissue. When MPS exceeds MPB, a positive muscle net protein balance and, by extension anabolic environment, occurs. In healthy adults, MPS is generally the more responsive variable and is the primary mediator of muscle net protein balance (7) and long-term changes in muscle mass (9). However, MPS is also important for replacing old, damaged, and mutated tissue proteins to maintain muscle quality (10). Thus, the optimal stimulation of MPS ultimately influences the mass and quality of skeletal muscle, which may impact a variety of health and/or performance related factors including glucose utilization (1), resting and activity energy expenditure (11), and disease risk and mortality (12).

The dietary strategy of intermittent fasting (IF) has become a topic of interest as an avenue to improve health (13, 14) and is often divided into three subclasses: alternate-day fasting, whole-day fasting, and time-restricted eating (TRE) (14). Alternate-day fasting involves alternating between *ad libitum* feeding days and very low energy intake (e.g., a single meal containing ~25% of daily calorie needs) or complete fasting days. Whole-day fasting typically consists of 1–2 days of either complete abstinence from calories or severe restriction on fasting days plus *ad libitum* eating on the other days. Finally, TRE, which arguably is the “mildest” form of IF, consists of restricting one's eating window to a certain number of hours per day often ranging from 4 to 8 h (14) with a suggested frequency of 1–3 meals (13). Thus, these IF strategies ultimately have a marked influence on the availability of postprandial dietary AA to support MPS and insulin to attenuate MPB.

Many of the health promoting effects of IF are mediated by its effectiveness to induce weight loss (15). For example, when IF is compared to controls with no intervention it generally results in weight loss (16, 17), although when compared to continuous energy restriction it is not superior in this outcome (18). By first principles, this suggests that IF may be an elementary means of inducing energy deficiency with no further diet modifications, which may in the short term enhance dietary adherence (19). This proposition is supported by the observation that skipping meals for up to 12 weeks is not compensated for by an increase in energy intake at subsequent meals consumed *ad libitum* (20). Additionally, 18 h compared to 12 h fasting has demonstrated significantly lower ghrelin levels, which could contribute to the reported reduced desire to eat and increased fullness over a 24 h period (21). Thus, as reduced energy availability can influence MPS rates (22, 23), IF strategies would need to consider the impact of total energy intake as a potential confounder contributing to the postprandial regulation of muscle protein turnover.

The following discussion outlines the current understanding of muscle protein metabolism in relation to the anticipated effect of IF as a dietary strategy on muscle mass and remodeling.

## Nutritional regulation of muscle protein breakdown

The breakdown and removal of muscle proteins is regulated by the ubiquitin-proteosome, calpain, and autophagy systems. While some benefits of IF are suggested to be mediated by increased autophagy (24), induction of this system with short term fasting (i.e., up to 36h) is not readily apparent in human skeletal muscle, unlike with exercise (25, 26). In contrast, the ubiquitin-proteosomal and calpain systems are the primary systems regulating nutrient and contraction-induced changes in MPB in humans (7) and therefore will be the primary focus of the present review. MPB is sensitive to feeding indirectly via the nutrient (i.e., carbohydrate and/or AA)-induced release of insulin from the pancreas (27). Maximal reductions in MPB require only modest elevations in plasma insulin concentrations (i.e., ~15–30 mU/L) (6, 28), which can be stimulated with a modest carbohydrate or protein intake (i.e., ~20–30g) (29, 30). Thus, the postabsorptive state when insulin is low is characterized by the highest rates of MPB to supply free AA, which are primarily “stored” in skeletal-muscle proteins (31), for other tissues (32–34) and as gluconeogenic precursors (31, 35, 36). This enhancement in MPB is demonstrated both with an overnight (~10 h) fast (31, 35) and prolonged (60–72 h) fasting (36–38). Given that IF typically involves a relatively prolonged fasting period (i.e., ≥16 h) as a primary means to reduce systemic insulin and promote lipolysis, MPB would be greater over a 24 h period with IF as compared to more typical meal feeding (i.e., 3–5 meals over ~16 h postprandrial period). With the contraction-induced anabolic stimulus of resistance exercise there is an increase in MPB, although this primarily serves to provide AA precursors to support MPS in the fasted state (39, 40). Thus, resistance exercise may help retain muscle mass with IF by attenuating the negative muscle protein balance of fasted, rested muscle. However, the exercise-induced increase in MPB is completely ablated with exogenous AA (41), highlighting an important role for dietary AA to support muscle anabolism via attenuated catabolism as well.

## Nutritional regulation of muscle protein synthesis

Dietary AA are the primary stimulators of and precursors for the synthesis of new muscle proteins (42). The equivalent of ~0.25 g/kg of leucine-enriched dietary protein in a single meal generally provides a saturating dose of AA for the postprandial stimulation of MPS (43–45), which persists for up to 6 h with the ingestion of whole foods (e.g., egg, beef and dairy proteins) (46–52). Importantly, after attainment of peak MPS (i.e., ~1.5–3h after protein feeding) (46, 47, 49, 51, 53), MPS gradually reverts back to basal levels even in the presence of sustained plasma aminoacidemia (54, 55). This is referred to as the “muscle full” effect (56) and demonstrates that there is a refractory period following ingestion of a protein bolus with the MPS pathway not able to be stimulated sequentially for ~3–5 h. Resistance exercise can prolong this postprandial muscle protein synthetic response (i.e., >5 h) (particularly of the myofibrillar fraction) (57, 58), although the maximal stimulatory protein dose is similar to what is sufficient at rest (i.e., ~0.3 g/kg) (45). There is some evidence that energy deficiency may increase the acute meal protein intake required to maximize MPS (22, 59) with estimates of ~0.4–0.5 g/kg being potentially sufficient (45). While protein and AA may have an insulinogenic effect (29), insulin only has a permissive effect for supporting maximal rates of MPS at rest and after exercise (29, 30, 60). Thus, manipulating the amount and timing of dietary AA ingestion represents the most important nutritional variable to optimize MPS.

## Nutritional regulation of amino acid oxidation

AA oxidation is generally low after an overnight fast but can increase with the duration of the fast (i.e., up to 3 d) (37), which during a period of acute starvation would contribute to a negative whole body (61) and muscle protein balance (38). While meal protein ingestion initiates a normal postprandial increase in AA oxidation (62), dietary AA consumed in excess of their ability to be incorporated into new body (especially muscle) proteins are further irreversibly oxidized and their nitrogen excreted (43–45). It has been suggested that the protein dose required to enhance whole body anabolism may be substantially greater than that required at the level of the muscle (63, 64). Accordingly, it is theorized that AA may be sequestered in splanchnic tissue (primarily the gut) to be later broken down and made available for synthesis of other tissues including muscle (63), although this has yet to be demonstrated. Thus, it is arguably more beneficial to consume acute meal protein intakes that maximize MPS yet minimize AA oxidation in order to optimize the daily dietary protein efficiency. In support of this notion, a recent study (21) comparing a 6 h feeding window with 3 meals to a 12 h feeding window with three meals (protein intake of ~0.3 g/kg per meal), the 6 h feeding window had significantly increased rates of 24 h protein oxidation by ~13 g/d (~85 vs. ~71 g/d).

## Discussion

**Figure 1:**
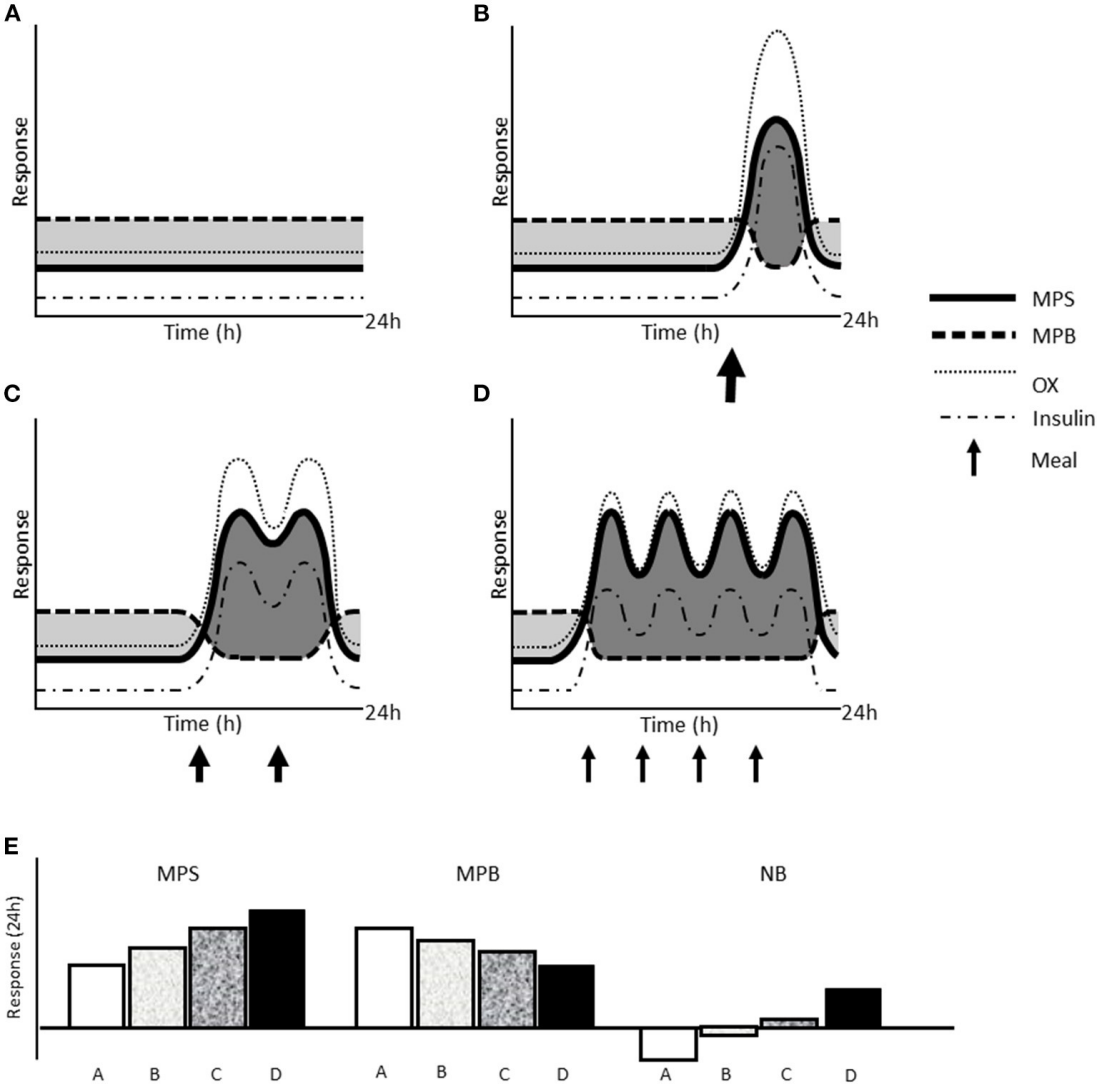
Schematic representation of the effect of different types of IF on acute muscle protein kinetics. Changes in muscle protein synthesis (MPS) and breakdown, amino acid oxidation (OX), and circulating insulin concentration over 24 h at rest with whole day fasting **(A)**, TRE with one meal **(B)**, TRE with two meals over 6–8 h **(C)**, and an unrestricted eating window (~16 h) with balanced meal pattern **(D)**. Size of arrows reflects the relative protein and energy content of each meal. Shaded dark gray areas indicate time in net positive protein balance and light gray areas indicate time in net negative protein balance, which are summarized as net 24 h response in MPS, MPB, and net muscle protein balance (NB; MPS—MPB) **(E)**.

Research on IF is growing exponentially with ~34 and ~45% of the >600 and >200 references since 2010 occurring in the past calendar year for the search terms “intermittent fasting” and “time-restricted eating,” respectively (source: Pubmed^®^ accessed December 11, 2020). A current limitation to the field of IF research is that no study, to the best of our knowledge, has measured muscle protein kinetics with alternate-day fasting or TRE. However, information may be gleaned from studies investigating the impact of daily feeding pattern on protein metabolism. For example, consuming a balanced pattern of moderate protein-containing meals (i.e., 3–4 meals at ~0.25–0.3 g/kg per meal) supports greater rates of myofibrillar and mixed muscle protein synthesis (65, 66) as well as whole body net balance (67) at rest and during recovery from resistance exercise in energy balance as compared to larger less frequent meals or in a skewed distribution (i.e., majority of protein in a single meal). These longer acute trials (i.e., 12–24 h) support the “muscle full” concept (56) that is exemplified by a maximal muscle protein synthetic response to acute protein ingestion (68). Collectively these acute studies support the concept that meal feeding pattern, irrespective of total protein intake, can influence whole body and muscle protein remodeling with large protein-containing meals stimulating postprandial AA oxidization rather than muscle tissue synthesis (Figure 1). Thus, based on the acute research to date, we argue that the lost opportunity for AA-induced MPS with more feedings may not be compensated for with fewer feedings at higher doses, as what is likely to occur with IF.

To our knowledge, no studies have examined whether adaptations in MPB, MPS, and AA oxidation take place over time to an IF protocol. Available literature suggests that following an overnight fast, the first meal demonstrates a similar MPS response to other meals (65), including those preceded by a large protein containing mixed-meal 4 h prior (44). Also, no adaptation is observed in the MPS response after 7-days consuming a skewed distribution of daily protein intake (66), drawing into question whether adaptation may occur with prolonged fasting and/or a chronically altered dietary protein intake pattern such as with IF. Therefore, while we cannot discount that the MPS response may be greater with a meal that breaks a prolonged fasting window and/or that MPB may adapt to a lower set point with chronic IF, there is currently little evidence to support this thesis.

Randomized control trials analyzing the effect of IF on fat free mass (FFM) demonstrate similar (19, 69–81) outcomes compared to controls. As IF often results in negative energy balance and weight loss (16, 17), when IF is compared to continuous energy restriction some systematic reviews suggest similar (82) or enhanced (83) preservation of FFM. The divergence in some of these results may be due to the differences in the types of IF or the self-selected meal frequency by research participants. As discussed above, there is a broad range of IF protocols and those which result in fewer meals (e.g., whole-day) would have greater effects than those with more meals (e.g., TRE). It is also important to note that the length of the studies to date may not have been sufficient to elucidate differences in FFM given the sensitivity of body composition measurement modalities used and their ability to detect changes over short (i.e., ≤12 weeks) interventions (84–86). Of note is a relative large recent study (*n* = 116 adult participants) that reported reductions in appendicular FFM by dual-energy X-ray absorptiometry with TRE over 12 weeks (87), which may be more representative of skeletal muscle mass than total FFM (88). Many of the studies mentioned above prescribe variations of IF as the independent variable but do not explicitly control dietary intake (19, 69, 70, 72–78, 81, 89, 90) and/or physical activity (19, 69–72, 75–78, 80, 89, 90), the latter of which is important to consider given that spontaneous physical activity may be modified by restricted eating (91) and can also influence the sensitivity of skeletal muscle to dietary AA (92). When IF is coupled with the potent anabolic stimulus of resistance exercise, a systematic review (93) observed no significant differences in FFM outcomes when compared to those resistance training with a normal diet. However, given the normal diet group also did not experience gains in FFM, as would be expected, the length (i.e., 4–8 weeks) of the included studies may also not have been adequate to reliably measure changes in FFM. It has been proposed that interventions >8 weeks are required for reliable FFM differences to become apparent with resistance training (94). In fact, a recent study suggests that resistance training-induced gains in FFM over 12 weeks are enhanced with a balanced as compared to a skewed daily protein distribution in healthy young men despite consuming a moderate (i.e., 1.3–1.45 g/kg/d) protein intake (95), which could be lower than that which would maximize growth (96, 97). Collectively, research to date evaluating the impact of IF on changes in body composition in young adults with and without prescribed exercise is equivocal. Therefore, it is important to acknowledge that the hypothesis of IF having consequences for muscle mass in particular may be complex. Based on our current understanding of acute muscle protein metabolism, the potential effect of IF may be small relative to other lifestyle related variables (e.g., total protein intake and exercise) but could be meaningful when extrapolated over time. However, we acknowledge that acute measures of muscle protein metabolism in laboratory settings may be oversimplified and their relationship to muscle mass and/or muscle quality need further investigation (9).

A limitation in evaluating the impact of IF on muscle mass and function is the overreliance on whole body estimates of FFM, which have been questioned as to their ability to specifically delineate skeletal muscle mass given they include substantial organ and non-muscle lean tissue (98, 99). While including additional outcomes such as appendicular lean mass, muscle thickness, or cross-sectional area, and/or fiber characteristics would help address the consequences of IF on muscle mass, characterizing changes in muscle protein turnover has been suggested to be an effective means to “predict” the direction of change in muscle mass over time, especially if measured over days (100, 101). Therefore, future research should include muscle specific outcomes (e.g., measures of mass and/or function) in chronic, controlled diet trials and/or measures of muscle protein turnover in acute trials to more clearly establish the impact of IF on skeletal muscle quality.

If the hypothesis of more protein feedings per day being optimal for mass and remodeling based on the acute literature is true, IF may represent a dietary conundrum for some populations. While IF is often employed to reduce feeding intakes, restrict total energy intake, and maintain a low insulin profile to help mobilize and metabolize endogenous fat (13, 14), based on our current understanding of the acute, nutritional regulation of muscle protein turnover it seems antithetical to what would presumably optimize muscle protein synthesis and net muscle protein balance (as summarized in Figure 1). Critically, populations who may experience a level of “anabolic resistance” to dietary protein, such as sedentary obese (102) and/or older adults (103), may be further susceptible to the suboptimal muscle protein turnover and anabolic environment borne of IF. For example, older adults who consume a balanced daily protein intake and/or consume a greater number of meals containing adequate protein ingestion generally have greater leg lean mass and muscle strength (104). There is also evidence that reduced energy availability, which often occurs in tandem with IF (16, 17, 20), increases the per meal protein intake required to maximize muscle protein synthesis (22, 23). Thus, while this would ostensibly favor larger protein meals that may be characteristic of TRE in particular, it does not preclude the need to consume protein more frequently, which would ultimately also help meet the higher recommended daily protein intakes that enhance muscle and FFM retention with weight loss (59, 105). Finally, performance populations such as athletes and military personnel may also be concerned with the quality of retained muscle/FFM with or without targeted weight loss (59), which would be important considerations for future research.

If the acute effects of IF lead to detrimental long-term outcomes for muscle, whole-day, and alternate-day fasting would have the greatest consequential effect on muscle mass and remodeling. This is due to the prolonged period with greater MPB and lower MPS compounded by the greater energy deficient state likely to occur (107) relative to TRE (108). In consideration of TRE, fewer meals would likely have a greater negative impact on muscle protein turnover (Figure 1). If TRE were to be employed, the hypothesis to improve muscle mass and remodeling suggests that protein intake should be consumed at a daily intake of at least 1.6 g/kg and into the number of meals that the feeding window allows separated by 3–5 h.

In conclusion, while IF may represent an option for a variety of populations to promote fat loss and improve aspects of metabolic health, additional research needs to focus on the impact of meal frequency on the quantity and quality of muscle mass. Inasmuch as IF may be purported as the enemy of body fat, future research must ensure this is not also the case for muscle. From our current understanding of muscle protein metabolism and taking a “muscle-centric” view for diet, we highlight that current acute evidence suggests IF may represent a counterproductive strategy to optimize muscle mass and, as far as protein turnover can remodel old/damaged proteins, muscle quality. Thus, studies that concurrently measure muscle protein metabolism and muscle mass and function will be instrumental in resolving these issues.

## Data Availability Statement

The original contributions presented in the study are included in the article/supplementary material, further inquiries can be directed to the corresponding author/s.

## Author Contributions

EW and DM wrote and revised the manuscript. Both authors read and approved the final version.

## Conflict of Interest

The authors declare that the research was conducted in the absence of any commercial or financial relationships that could be construed as a potential conflict of interest.
